# Biased cultural transmission of a social custom in chimpanzees

**DOI:** 10.1126/sciadv.ade5675

**Published:** 2023-02-15

**Authors:** Edwin J. C. van Leeuwen, William Hoppitt

**Affiliations:** ^1^ Animal Behaviour and Cognition, Department of Biology, Utrecht University, Utrecht, the Netherlands.; ^2^ Centre for Research and Conservation, Royal Zoological Society of Antwerp, K. Astridplein 26, B 2018 Antwerp, Belgium.; ^3^ Department of Comparative Cultural Psychology, Max Planck Institute for Evolutionary Anthropology, Deutscher Platz 6, 04103 Leipzig, Germany.; ^4^ Department of Biological Sciences, Royal Holloway, University of London, Egham, UK.

## Abstract

Cultural transmission studies in animals have predominantly focused on identifying between-group variation in tool-use techniques, while immaterial cultures remain understudied despite their potential for highlighting similarities between human and animal culture. Here, using long-term data from two chimpanzee communities, we tested whether one of chimpanzees’ most enigmatic social customs—the grooming handclasp—is culturally transmitted by investigating the influence of well-documented human transmission biases on their variational preferences. After identifying differences in style preferences between the communities, we show that older and dominant individuals exert more influence over their partners’ handclasp styles. Mothers were equally likely to influence their offspring’s preferences as nonkin, indicating that styles are transmitted both vertically and obliquely. Last, individuals gradually converged on the group style, suggesting that conformity guides chimpanzees’ handclasp preferences. Our findings show that chimpanzees’ social lives are influenced by cultural transmission biases that hitherto were thought to be uniquely human.

## Teaser

Chimpanzees learn from dominant group members how exactly their group-specific social custom of “handclasping” is to be performed.

## INTRODUCTION

The past few decades have witnessed an accumulation of evidence in favor of the existence of nonhuman animal (henceforth “animal”) culture (*1*–*4*), especially in the realm of tool manufacturing and tool use (*5*–*7*). Ranging from insects to whales (*1*), animals form (local) cultural traditions by means of social learning—the process in which individuals acquire information about their environment by observing or interacting with their conspecifics or the consequences of their conspecifics’ actions (*8*). Social learning is a prerequisite feature in the ethological concept of culture, which can be defined as “all group-typical behavior patterns, shared by members of animal communities, that are to some degree reliant on socially learned and transmitted information” (*9*). Especially when social learning is selective such that it ensues in certain contexts (e.g., in times of uncertainty) or favors certain individuals as models (e.g., experts or dominants) does it optimize the quality of extracted information (*10*, *11*). Such contingencies are known as social transmission biases (*12*) or social learning strategies (SLSs) (*10*) and lie at the heart of cultural evolutionary dynamics in humans (*13*, *14*).

Recent studies have aimed to identify similar SLSs in animals. For instance, capuchins (*Cebus capucinus*) were found to be highly payoff-biased in their social learning (*15*), while vervet monkeys (*Chlorocebus pygerythrus*) selectively learned (new) food-processing techniques from high-ranking individuals (*16*, *17*). In conjunction, an impressive body of evidence has been compiled in which animal SLSs are presented (*18*). This cross-species tracking of SLSs is important for identifying the evolutionary origins of human culture and sketching informed scenarios in which such learning biases may have evolved. One important caveat, however, is that the SLS endeavor focuses almost entirely on tool usage and extractive foraging capabilities, whereas a large part of (human) culture exists in the social domain. Human culture permeates the very fabric of our social interactions, ranging from our greeting mannerisms and interindividual space preferences to turn-taking in conversations and norm adherence (*19*–*22*). This may be true for animals as well (*23*). For instance, song birds and cetaceans may develop vocal dialects that culturally evolve to become markers of group identity (*24*, *25*) while at the same time affecting the social structures they live in (*26*). If we wish to understand cultural dynamics comprehensively, we need to investigate SLSs within this essential domain of human and animal culture (*23*).

**Fig. 1.**
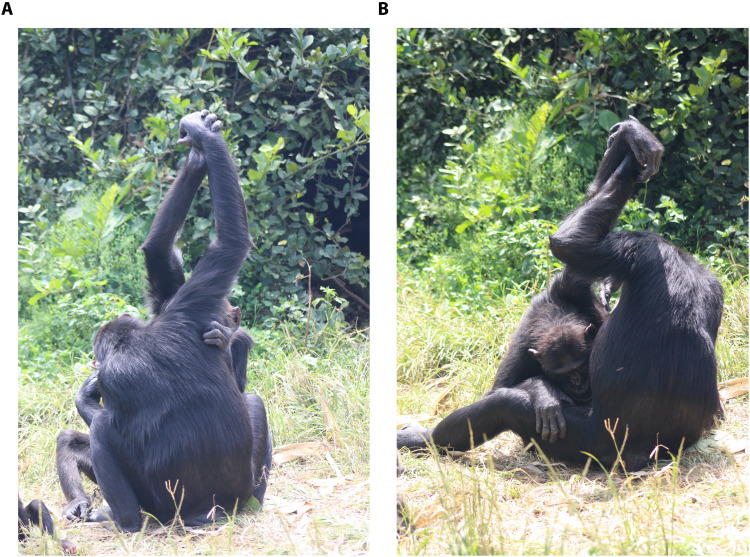
Chimpanzees at the Chimfunshi Wildlife Orphanage Trust (Zambia) engaging in grooming handclasps. The chimpanzees use different styles of handclasping, for instance, (**A**) palm-to-palm style and (**B**) palm-to-wrist style.

Here, we investigate whether the social transmission of chimpanzees’ grooming handclasp (GHC) preferences follows nonrandom pathways to identify animal SLSs in the sociocultural domain. The GHC is the first described social custom in chimpanzees (*27*) and remains one of the most convincing examples of social culture in animals (*28*, *29*). In a handclasp, two chimpanzees simultaneously extend one of their arms overhead and clasp each other’s extended hand at the palm, wrist, or forearm while typically grooming each other with the other arm (Fig. 1).

Initially, the handclasp was interpreted as culture based on the argument that some chimpanzee populations engaged in it, while others, including long-term monitored populations, did not (*27*, *30*). Later, with an increasing number of populations reported to handclasp over time, the argument for culture refound its strength in the observation that different communities show different preferences for which part of their arm to clasp onto (*29*, *31*). However, an important outstanding question remains: How do these handclasp style preferences transmit within groups?

In our study, we focus on two major transmission biases pivotal to human culture: the majority bias and the dominance/prestige bias (*32*, *33*). In humans, there is ample evidence that social behaviors are selectively copied from majorities (*34*–*36*) and from people who enjoy either a form of dominance or a certain level of prestige—an accrued status of expertise due to expressions of excellence in a valued domain (*33*). It is thought that both the majority and dominance/prestige biases have evolved owing to their probabilistic power to indicate information quality (*32*, *33*). Specific to the social compared to the material domain, however, majority and/or dominance bias may be expected to nurture social capital in the form of strategic (future) engagement by group members via homophily (*36*, *37*), for instance, in contexts where coalitionary support or cooperative commitment could confer benefits (*38*). In animals, the majority bias has been identified in the domain of foraging and mate choice (*39*–*41*). Similarly, in chimpanzees, there have been two documentations of dominance- and prestige-biased transmission in the context of experimentally seeded problem-solving techniques (*42*, *43*). [Strictly speaking, the dominance and prestige biases have been distinguished on the basis of the former being forcefully exercised and the latter being freely conferred (*33*).] However, in the social domain, we are currently unaware of the existence of animal SLSs, neither in the form of a majority bias nor in the form of dominance/prestige biases.

Here, by applying tailored Bayesian analyses to a longitudinal database (2007–2019) on GHC variations in two communities of chimpanzees (*n* = 76), we investigate whether (i) the original intercommunity variation in style preferences can be identified [see (*29*)], (ii) higher-ranked individuals exert more influence over the transmission of style preferences compared to lower-ranked individuals (i.e., dominance bias), and (iii) maturing individuals increasingly converge in their variant preference on the majority preference (i.e., conformity bias). Given that age correlates with dominance (and prestige) in chimpanzees (*43*), we additionally investigated the effect of age on exerting influence on the handclasp style preferences. In light of the documented biases in the technological domain [at least the dominance bias (*42*), for the conformity bias in chimpanzees, mixed evidence exists (*44*–*47*)], we hypothesized that the chimpanzees would transmit their handclasping practice according to the respective biases [although see (*48*)]. We investigated our hypotheses using a within- and between-subjects design, sampling naturally occurring handclasp events during social interactions in semiwild chimpanzees (*Pan troglodytes schweinfurthii*) in the Chimfunshi Wildlife Orphanage Trust, Zambia (Fig. 1). We conducted all-occurrence sampling (*49*) on 71 handclasping chimpanzees of two resident groups and measured the style usage during handclasping for each individual at the resolution of five mutually exclusive arm regions (*50*).

## RESULTS

### Group preferences

The results support the hypothesis that there are underlying group preferences, after considering sampling error at the level of individuals (Table 1). The palm style was the most popular variant in both groups, but this preference was more pronounced in group 2 than in group 1. In group 1, chimpanzees were more likely to use the wrist and elbow variants.

**Table 1.** Bold cells indicate strong evidence for a difference in handclasp preference between the groups, with a positive difference (right column) in favor of group 1 and a negative difference in favor of group 2. HPDI, highest posterior density interval.

*k*	Variant	Mean of posterior [95% HPDI]		
		Group 1 ${\hat{p}}_{1k}$	Group 2 ${\hat{p}}_{2k}$	Difference ${\hat{p}}_{1k}-{\hat{p}}_{2k}$
**1**	**Wrist**	**0.341**	**0.128**	**0.213**
		**[0.239, 0.449]**	**[0.089, 0.169]**	**[0.102, 0.328]**
**2**	**Elbow**	**0.013**	**0.001**	**0.011**
		**[0.003, 0.025]**	**[0.000, 0.002]**	**[0.002, 0.023]**
3	Forearm	0.024	0.009	0.015
		[0.009, 0.042]	[0.004, 0.015]	[−0.0021, 0.034]
4	Other	0.008	0.003	0.004
		[0.002, 0.015]	[0.001, 0.006]	[−0.003, 0.013]
**5**	**Palm**	**0.615**	**0.859**	**−0.244**
		**[0.497, 0.731]**	**[0.813, 0.901]**	**[−0.369, −0.119]**

### Transmission biases

#### Dominance, age, and sex bias

**Fig. 2.**
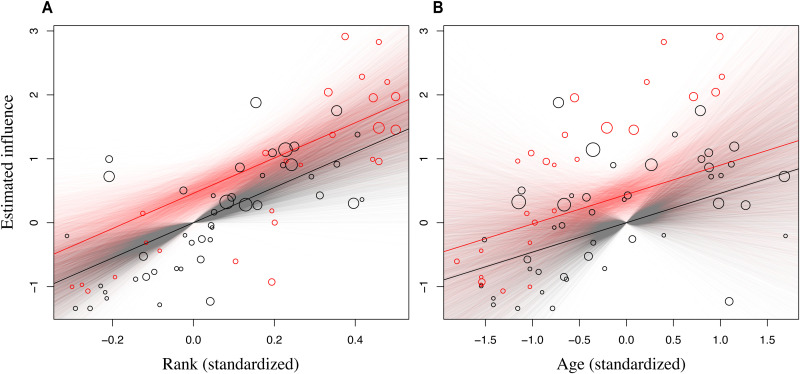
The styles by which the chimpanzees engaged in the grooming handclasp could be linked to sociodemographic features. The estimated influence on handclasp style preference depicted (**A**) across the rank continuum and (**B**) across the observed age range. The sizes of the circles (raw data) are proportional to the number of observations, the solid lines represent the averaged regressions, and the transparent lines depict all the posterior samples. Red, males; black, females.

There is strong evidence that higher-ranking chimpanzees had a stronger influence than lower-ranking chimpanzees, with β_rank_ = 2.78 {95% highest posterior density interval (HPDI) = [0.602, 5.09]}, corresponding to the highest-ranking individual in a group (rank = 1) being an expected exp(2.78) = 16.1× more likely to influence the choice of variant than the lowest rank (rank = 0) (Fig. 2A). Likewise, there was reasonable evidence that age had an effect, with β_age_ = 0.440 (95% HPDI = [0.031, 0.858]), corresponding to an increase of 1.56× influence with 1 SD increase in age (Fig. 2B). As expected in chimpanzees, rank and age were correlated (Pearson’s product-moment, *r* = 0.45, *P* < 0.001), but given that both measures were assessed in the same model context, they represent coexisting effects with any influence of collinearity captured in the uncertainties (95% credible intervals) for the effects of these variables. In contrast, there was little evidence that sex had an effect on influence, β_sex_ = 0.460 (95% HPDI = [−0.601, 1.515]; see Fig. 2, A and B). For more details, see the Supplementary Materials.

#### Maternal effects on style preference

We expected that young chimpanzees have a style preference that starts close to their mother’s preference and over time converges on the group mean (*29*, *48*). The mean of the posterior distributions for the maternal influence on chimpanzees’ style preferences was *m*_≤8_ = 0.259 (95% HPDI = [0, 0.728]) and *m*_>8_ = 0.425 (95% HPDI = [0, 0.917]). The width of the 95% HPDIs shows that we cannot estimate *m* with great precision for either age category (see fig. S1); however, some inferences can be drawn. First, *m*_≤8_ − *m*_>8_ is estimated at −0.166 (95% HPDI = [−0.849, 0.439]), indicating that there is no evidence that the maternal influence is stronger on individuals aged 8 years or younger. The posterior sample for *m*_≤8_ also reveals that the most likely value is *m*_≤8_ = 0 and that *m*_≤8_ = 1 has a low posterior density (fig. S1). This shows that the data are not consistent with only mothers influencing the preferences of their offspring, indicating that other group members have a substantial influence on young chimpanzees’ handclasp style preferences. This notion is corroborated by the fact that the number of unique handclasp partners steadily increases with age (fig. S2).

#### Convergence toward the majority

**Fig. 3.**
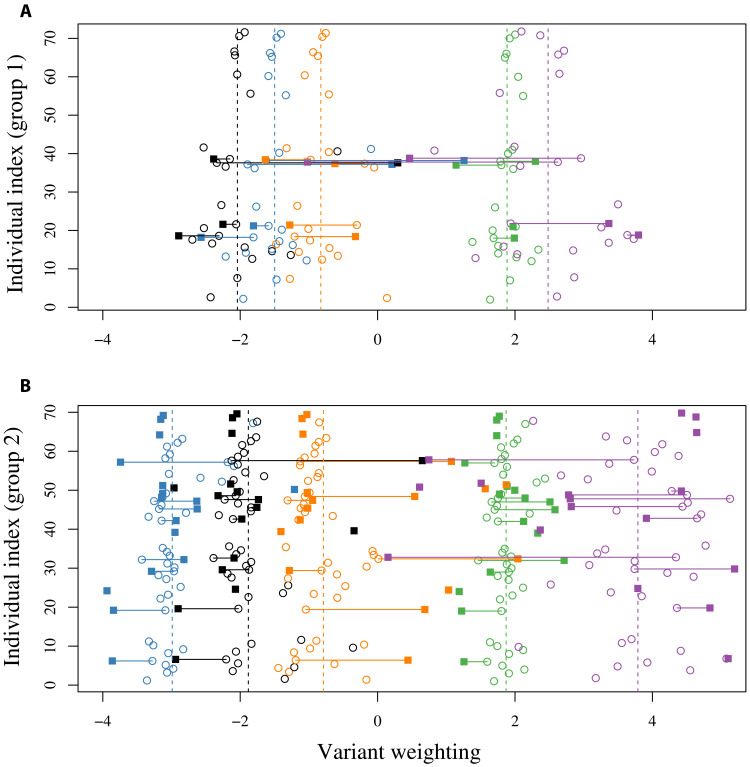
The chimpanzees have group-specific handclasp preferences, with younger chimpanzees showing more variation than older chimpanzees. The estimated preferences for each option (green, wrist; black, elbow; yellow, forearm; blue, other; purple, palm) for (**A**) group 1 and (**B**) group 2. Dashed lines show the estimated group preferences μ*_jk_*, whereas points represent individual preferences. Open circles are the handclasp style preferences for individuals >8 years old, and filled squares are the preferences for individuals ≤8 years old. Lines connect the same individuals as they age. The points for younger individuals are more widely scattered around the group means than those for older individuals.

There is strong evidence that individual preferences tended to differ more from the group mean among younger individuals, indicating that, over time, chimpanzees converge on the group preference after an initial phase of style exploration (Fig. 3; for the raw proportions of each option, see fig. S3). σ*_W_*, the variance in *W_ijk_* within each group from the group means μ*_jk_*, was estimated to be σ_*W*,>8_ = 1.02 (95% HPDI = [0.81, 1.25]) for chimpanzees older than 8 years and σ_*W*,≤8_ = 2.37 (95% HPDI = [1.58, 3.24]) for chimpanzees younger than 8 years. The difference in variances was estimated to be σ_*W*,≤8_ − σ_*W*,>8_ = 1.35 (95% HPDI = [0.53, 2.22]) with *p*(σ_*W*,≤8_ > σ_*W*,>8_) = 1.00. Note that we also tried the split ≤10/>10 and found a less marked difference in σ_*W*≤10_ > σ_*W*>10_, suggesting that preferences may already have stabilized by age 9 or shortly afterward.

## DISCUSSION

This study sought to investigate whether nonhuman animals use social learning strategies (SLSs) in the transmission of their sociocultural practices. While selective (social) learning is regarded as a key property of human culture and its adaptive value (*51*, *52*), comparatively, little is known about its presence in other species, thereby hampering an evolutionary account of its emergence. We find that chimpanzees have group-specific preferences for executing a social custom and identify SLSs in the transmission of their handclasp custom in the form of majority convergence, dominance bias, and age effects. Young chimpanzees used a larger variety of handclasp styles compared to older chimpanzees, which indicates that the chimpanzees gradually converge on the group-specific style preference [see (*53*) for a similar finding regarding the adoption of seed-extraction techniques in capuchin monkeys]. Such a convergence may be facilitated by a conformist bias, in which individuals disproportionately adopt the majority strategy (*10*, *54*). Animals have been found to conform under some conditions (*39*–*41*), although the evidence still requires validation (*55*, *56*), and the findings for chimpanzees are at best equivocal (*47*, *57*, *58*). In the current study, however, a less (cognitively) demanding majority influence may have shaped the chimpanzees’ style adoptions, namely, majority-biased transmission or copy the majority, in which the presence of a more homogeneous (older) generation of handclaspers favors the odds of adopting the majority strategy without a selective majority bias (*59*, *60*). With more targeted observations and experiments, the exact underlying mechanism may be identified (*39*).

The observed group differences in handclasp style preferences may alternatively (or simultaneously) be explained by the identified dominance bias, in which dominant individuals exert substantial influence over the styles used by their partners in subsequent bouts. Chimpanzees have been found to preferentially learn from dominants in reward-based experiments with artificial tools (*42*, *43*). Here, we report evidence of an important SLS (*18*) in a spontaneously occurring custom in the social domain in nonhuman animals. Selectively adopting information from dominant individuals may provide an effective way of navigating the complexities of the social world (*61*, *62*), especially given that dominance may overlap with prestige (*33*) and in conjunction correlate with social benefits in the form of social acceptance, privileges, and preferred social bonding (*33*, *63*). In this light, it is worth considering to what extent SLSs may differ across domains. For instance, in the realm of material cultures (e.g., tool use for nut cracking), it may be adaptive to selectively copy the technique from the most successful individual, whereas in the realm of social cultures, copying may be guided more by (strategic) expectations of social benefits like coalitionary support. A recent case study reported that a female chimpanzee copied a local social tradition among the resident females (i.e., “crossed-arm walking”) immediately upon immigrating into a new group, which the authors quantitatively connected to her subsequent relatively quick social integration (*37*). Knowing that resident female chimpanzees in the wild typically respond agonistically to female immigrants (*64*), such selective copying by the immigrant may confer substantial fitness benefits, possibly mediated by the mechanism of homophily, in which individuals prefer similar over dissimilar others (*36*). To advance our understanding of cultural dynamics, future studies could further expose the effects of social attributes like dominance on sociocultural behavior in animals, for instance, by investigating the extent to which culture itself affects the reach of influential individuals (*23*, *26*). A plausible scenario could posit that the way that societies are organized in terms of social connectedness substantially affects the extent to which information—be it practically (e.g., tool use) or socially (e.g., nurturing bond partners) functional—transmits through them. Here, the hypothesis would be that tightly knit social fabrics enhance the spread of information (*65*). A further high-potential avenue for future research, particularly in the handclasp context, comprises the study of the exact mechanism by which animals copy. Recent correspondences have revisited the question of whether animals can copy the specific forms of behavior from others given its presumed relevance to cumulative culture (*66*, *67*). In the current study, because of the different focus, we remain agnostic about the mechanism of social transmission. The observed patterns of form convergence, however, suggest that the chimpanzees not merely adopted one handclasp style passively resulting in group-specific preferences but (over time) changed their specific forms of handclasping in response to interacting with (and/or observing?) others (also see fig. S4). To understand how exactly the chimpanzees adopt the handclasp styles of others, higher-resolution data including individuals’ observation records will be required (*55*).

Despite their importance in the acquisition of skills and ecologically relevant information (*68*, *69*), chimpanzee mothers were not obviously shaping the technique by which their offspring engaged in the GHC. Earlier findings show that mothers are in most instances the first handclasp partner of new adopters (*29*, *70*) and that even the specific technique of handclasping initially spreads within matrilineal family units (*48*). However, we show that mothers do not exert more influence over their offspring style adoption than other group members. Moreover, nonkin group members comprised a large portion of the subjects’ pool of handclasp partners, increasingly so for older chimpanzees. These findings indicate that the chimpanzees learned the specifics of their cultural practice not only vertically (i.e., from one generation to the next), which parallels the relatively slow-paced genetic adaptive process, but also horizontally (i.e., within generations), which is a pivotal characteristic of human cultural learning (*71*, *72*). In principle, horizontal social learning accelerates the function of culture as a second inheritance system by magnitudes (*73*). Hence, the SLSs reported here may provide important insights into chimpanzees’ latent capacities for developing the purportedly uniquely human form of culture, namely, cumulative cultural evolution (*74*, *75*). In particular, the conformist bias (*32*) and the dominance/prestige bias (*33*) may harness the potential of selective social learning to contribute to the accumulation of cultural knowledge (*18*). By showing that both these biases are implicated in chimpanzees’ adherence to a social convention, this report highlights the exciting possibility that cumulative cultural evolution, considered by many as one of the most prominent traits explaining the biological success of the human species (*76*), might have its roots in our evolutionary history.

## MATERIALS AND METHODS

### Subjects and data collection

Subjects included 71 handclasping chimpanzees (45 females and 26 males) from group 1 and group 2 (mean age + range: 15 years; 9 months; 5 to 33 years) at the Chimfunshi Wildlife Orphanage Trust (Zambia). Chimfunshi is a chimpanzee sanctuary in which chimpanzees live in large, forested enclosures ranging in size from 47 to 190 acres. Chimpanzees at Chimfunshi stay outside overnight and only come indoors for supplemental feeding between 1130 and 1330 hours. The chimpanzees of group 1 and group 2 do not have visual access to each other.

Data were collected using all-occurrence sampling (*49*) from elevated areas around the chimpanzee enclosures (e.g., holding facilities and anthills). Observations took place between May and July 2007, May and June 2010, May and June 2011, July and August 2017, and March and August 2019. During the observation sessions, all visible GHC bouts were both live-coded and video-recorded from an observation deck on top of the indoor facilities by three observers who recorded the identity and handclasp style. If identity and style could not be determined from live observation or video, the event was excluded from the analyses (*n* = 29). A total of 2049 handclasp events (thus including 4098 style productions) were obtained. Reliability between the project lead (E.J.C.v.L.) and a secondary coder (KAC) was established by independently scoring 112 GHC styles from a random selection of videos (Cohen’s κ = 0.91) (*77*). Rank was determined per observation window by averaging the ordinal rank assessments as independently provided by three experienced (6+ years) caretakers. The study was approved by the Chimfunshi Research Advisory Board and conformed to the nationwide legal requirements as stipulated by the Zambia Wildlife Authority.

### Handclasp style variants

There are different variants of handclasp grooming that can be performed—the main ones being wrist, elbow, forearm, and palm—and other variants are grouped together as “other.” Different groups seem to have different traditions in terms of the variants of handclasp grooming that tend to be used (*28*, *29*, *78*), suggesting that they might learn these preferences from their interactions with others in their group, e.g., a chimpanzee forms a preference for the elbow variant after engaging in handclasps predominantly with chimpanzees that prefer the elbow variant. The aim of the statistical models presented in this study is to shed light on whether there is evidence that such social learning takes place and how it operates.

### Statistical modeling: Preference and influence

To model social learning, we estimated the handclasp variant preferences of individuals: how likely are they to choose to perform a particular variant, given that they are the one choosing the variant. This is complicated by the fact that we often cannot be certain who has chosen which variant is performed in any given interaction. When both subjects choose different variants, it is reasonable to assume that they have both performed according to their own preferences. However, when they both perform the same variant, there are three possibilities: (i) They both choose a variant according to their own preferences, and their choices happened to match; (ii) subject 1 chooses the variant according to their own preferences and influences subject 2 to perform the matching variant; or (iii) subject 2 chooses the variant according to their own preferences and influences subject 1 to perform the matching variant. We solved this problem by assessing the portion of the model that predicts the variant observed by each individual in a given handclasp as a function of the preferences of each of the subjects, allowing those preferences to be estimated from the data.

#### Modeling choice of variant and estimating individual and group preferences

The response variable consists of a pair of observations for each event, *e*; the variant chosen by subject 1 [denoted *G*1(*e*) for “groomer 1” in event *e*], *y*_*G*1(*e*)_; and the variant chosen by subject 2, *y*_*G*2(*e*)_. Both can take the discrete values 1 = wrist, 2 = elbow, 3 = forearm, 4 = other, and 5 = palm. The probability, *p_ijk_*, of individual *i* in group *j* choosing option *k*, given that they made the choice of variant, was taken to be

$$p_{ijk}=\exp(W_{ijk})/\sum_{l=1}^{5}\exp(W_{ijk})$$ (1)

where *W_ijk_* gives individual *i* in group *j*’s preference for option *k*, relative to option 1 (wrist), where *W*_*ij*1_ = 0, where 2 = elbow, 3 = forearm, 4 = other, and 5 = palm. Therefore, exp(*W_ijk_*) estimates how much more likely it is that individual *i* in group *j* will choose option *k* than they will choose option 1, where, for *k* = 2, 3, 4, 5

$$W_{ijk}∼N(\mu_{jk},\sigma_{W})$$ (2)

where μ*_jk_* is the group mean preference for option *k*, and σ*_W_* is the individual variation in preference within groups. μ*_jk_* and σ*_W_* were estimated from the data with vague priors: μ*_jk_*~*N*(0,1000), σ*_W_*~*U*(0,10).

This means that, for any given handclasp event, the probability of option *k* being performed by subject 1 was

$$P[y_{G1(e)}=k]=[1-i_{c}(e)]p_{G1(e)jk}+i_{c}(e)p_{G2(e)jk}$$ (3a)

where *P*1(*e*) is the identity (*i* and *j* combination) of subject 1 for event *e*, *P*2(*e*) is the identity of subject 2 for event *e*, and *i_c_*(*e*) is an indicator that takes the value of 1 in situation *c* (= *G*2 chooses the variant according to their own preference and influences *G*1 to perform the matching variant) and 0 otherwise. Thus, if *G*2 decides the variant for both subjects, the likelihood for *G*1’s choice reflects the preferences of *G*2. Likewise, for *G*2’s performed variant

$$P[y_{G2(e)}=k]=[1-i_{b}(e)]P_{G1(e)jk}+i_{b}(e)P_{G2(e)jk}$$ (3b)

where situation *b* is where *G*1 chooses the variant according to their own preference and influences *G*2 to perform the matching variant.

As noted above, if *G*1 and *G*2 perform different variants for event *e*, we know that we have situation (1) above, and thus, *i_b_*(*e*) = *i_c_*(*e*) = 0. However, if both *G*1 and *G*2 perform the same variant, situations (1), (2), and (3) are all possible, and thus, *i_a_*(*e*), *i_b_*(*e*), and *i_c_*(*e*) are imputed according to the model described below. Imputing this variable means that our inferences about individual variant preferences take into account our uncertainty about who chose the variant for each event.

The group-level model was parameterized such that μ*_jk_* estimated the preference for *k* = 2, 3, 4, 5 relative to the preference for *k* = 1 within each group (with μ_*j*1_ constrained to be 0 to avoid overparameterization). This makes direct comparison of group preferences difficult, since preference for *k* = 1 might vary between the groups. Consequently, we transformed estimates of μ*_jk_* such that they represent the expected proportions of each variant for an average individual from each group for every iteration of the Markov Chain Monte Carlo (MCMC) algorithm, ${\hat{p}}_{jk}=\exp(\mu_{jk})/[\sum_{l=1}^{5}\exp(\mu_{jl})]$, allowing us to compare the preferences of each group.

#### Assessing cultural transmission biases

The probability of each situation is modeled as follows:

(1) *G*1 and *G*2 choose independently

$$P[i_{a}(e)=1,i_{b}(e)=0,i_{c}(e)=0]=\exp(\beta_{a})/[\exp(\beta_{a})+I_{G1(e)}+I_{G2(e)}]$$ (4a)

(2) *G*1 chooses for both

$$P[i_{a}(e)=0,i_{b}(e)=1,i_{c}(e)=0)]=I_{G1(e)}/[\exp(\beta_{a})+I_{G1(e)}+I_{G2(e)}]$$ (4b)

(3) *G*2 chooses for both

$$P[i_{a}(e)=0,i_{b}(e)=0,i_{c}(e)=1]=I_{G2(e)}/[\exp(\beta_{a})+I_{G1(e)}+I_{G2(e)}]$$ (4c)

where *I*_*P*1_ and *I*_*P*2_ are the influence of *G*1 and *G*2, and β*_a_* is a parameter [prior β*_a_*~*N*(0,1000)] estimating the baseline likelihood that both subjects choose their own option. The influence for each individual was modeled as follows

$$I_{ij}=\exp(I_{\mathrm{RE},ij}+\beta_{\mathrm{age}}{\mathrm{age}}_{ij}+\beta_{\mathrm{sex}}{\mathrm{sex}}_{ij}+\beta_{\mathrm{rank}}{\mathrm{rank}}_{ij})$$ (5)

where age*_ij_* is the age of individual *i* in group *j* (standardized across groups); sex*_ij_* is 1 = male, 0 = female; and rank*_ij_* is rank normalized such that the highest-ranking individual = 1 and the lowest-ranking individual = 0. Note that age and rank varied within individuals across years but are denoted as constant here for simplicity. β_age_, β_sex_, and β_rank_ were coefficients estimating the effect of each of these variables on influence, with prior ~*N*(0,1000). Last, *I*_RE,*ij*_ was an individual-level random effect on influence, where *I*_RE,*ij*_~*N*(0, σ*_I_*), with σ*_I_* estimating the variance among individuals not accounted for by age, sex, and rank [vague prior: σ*_I_*~*U*(0,10)].

To assess the relative influence of mothers on their offspring’s style preferences (*29*, *48*), we tested whether relatively young chimpanzees have a preference that starts closer to their mother’s and over time converges on the group mean. To investigate this, we expanded the model described in Eq. 2 with

$$W_{ijk}∼N[(1-m)\mu_{jk}+mW_{M(ij)k},\sigma_{W}]$$ (6)

where *M*(*ij*) is the identity of the mother of individual *i* in group *j*, and *m* is the level of maternal influence, where 0 indicates that the mother has no more influence than any other member of the group and 1 indicates that only the mother has any influence over the preference of *i*. The model allowed parameter *m* to vary for individuals 8 years old or younger, *m*_≤8_, and those older than 8 years, *m*_>8_. If chimpanzees start with a preference more influenced by their mother and converge on the group mean, we expect to find evidence for *m*_≤8_ > *m*_>8_. In cases where the mother was not known or not present in the data, *W*_*M*(*ij*)*k*_ was imputed as *W*_*M*(*ij*)*k*_~*N*(μ*_jk_*, σ*_W_*), allowing for uncertainty in these values.

#### Assessing convergence toward the group-specific style preference

If chimpanzees converge on the group preferences for handclasp variant over time, we expect their preferences to differ when they are younger from when they are older and also to tend to be more different from the group mean preferences when they are younger. The above model was thus expanded such that preferences were estimated separately for chimpanzees when they were ≤8 years old and when they were >8 years old with σ_*W*≤8_ and σ_*W*>8_ estimating the variance from the group means in each age category. If chimpanzees converge on the group mean preference during their early years of handclasp grooming, we expect evidence for σ_*W*≤8_ > σ_*W*>8_.

#### Bayesian estimation

Bayesian estimation was accomplished using the MCMC methods using the JAGS sampler (*79*), via the runjags (*80*) and coda (*81*) packages in the R statistical environment (*82*). Vague (uninformative) priors were specified for all model parameters, with variances having a prior of ~*U*(0,10) and other parameters having a variance of ~*N*(0,1000). We ran 1000 adaptive iterations followed by a burn-in period of 1000 iterations, which was found to be sufficient for the convergence of chains. We ran 20 chains in parallel on separate computer cores, sampling the MCMC every 10 iterations, running enough to obtain a thinned sample of 100,000. This resulted in an effective sample size of at least 6000 for all β parameters and at least 25,000 for all σ and μ parameters. The mean of the posterior distribution is used to estimate parameter values with the 95% HPDI used to show the precision of estimates.

## Supplementary Materials

### This PDF file includes:

Supplementary Results Figs. S1 to S4
