# Supplementary material for: Biased cultural transmission of a social custom in chimpanzees
Source: Sci Adv. 2023 Feb 15;9(7):eade5675. doi: 10.1126/sciadv.ade5675 (PMC9931211; doi:10.1126/sciadv.ade5675)
Supplement: Supplementary file 1 — Supplementary Results Figs. S1 to S4 [file sciadv.ade5675_sm.pdf]

Science Advances
AAAS

Supplementary Materials for

Biased cultural transmission of a social custom in chimpanzees

Edwin J. C. van Leeuwen and William Hoppitt

Corresponding author: Edwin J. C. van Leeuwen, ejcvanleeuwen@gmail.com

Sci. Adv. **9**, eade5675 (2023)
DOI: 10.1126/sciadv.ade5675

This PDF file includes:

Supplementary Results
Figs. S1 to S4

Results

1. Influence model for investigating transmission biases

We modeled the influence of individual's style usage on subsequent style adoption. In an estimated 47.7% (95% HPDI= [42.3, 52.8]) of cases, one of the participants influenced the choice of the other (i.e., chooses which variant was to be used). The results suggest that individuals varied in the influence they had over the handclasp variant to be used, and that higher ranking and older chimpanzees tended to have more influence than lower ranking and younger chimpanzees (see *Main text*). Yet, there was a substantial amount of individual variance in influence not accounted for by dominance rank, age, and sex, with σ_I= 1.068 (95% HPDI= [0.525, 1.652]).

2. A model of social transmission

The results in the main text suggest that young chimpanzees may acquire variant preferences – typical of their group – as a result of interacting with others. As an initial estimate, the model suggests that such social experience would be acquired approximately before the age of 9 years, by which time individuals tend to have converged on the mean group preference. In addition, we find evidence that higher-ranked, older chimpanzees influence the variant performed by other individuals with whom they engage in handclasp grooming. In conjunction, this leads us to hypothesize that preferences are shaped when a handclasp is performed with an influential individual who influences the variant used in the interaction. For instance, a young low-ranking individual "A" engages in a handclasp with an older high-ranking chimpanzee "B". "B" has a greater preference for the wrist variant, and influences "A" into also performing the wrist variant. As a result, "A" becomes more likely to choose the wrist variant in the future.

We attempted to test this hypothesis by assessing whether young chimpanzees' (≤8 years old) preferences changed during each observation window. We formalized the hypothesis by expanding the model such that an individual ≤8 years old increased their preference for a particular variant they were influenced into performing by another

individual. The amount of change to W_{ijk} was parameterized as s_{inf} (inf = influenced). Subsequently, we allowed for the possibility that the preference might be increased in favor of the variant performed by the other participant regardless of whether they were influenced into performing the same variant, by amount s_{all}. We then rescaled W_{ijk} such that $W_{ij1} = 0$. Both s_{inf} and s_{all} had vague priors ~N(0,1000).

Since we only have (approximately) complete data within each observation period, this learning rule was applied within each observation period separately for the individuals who were ≤8 years old at the time, with a new starting preference estimated for each individual from the data for each period in which they were within the target age-range.

We estimated s_{inf}= 1.32 (95% HPDI= [-0.63, 3.19]) and s_{all}= -0.03 (95% HPDI= [-0.28, 0.20]), with $p(s_{inf} > 0)$= 0.920 and $p(s_{all} > 0)$= 0.408. Therefore, we have weak, suggestive evidence for an effect of s_{inf}. Note that s_{inf} is the effect of "influenced" GHCs over and above that of "uninfluenced" GHCs (s_{all}). This suggests that the changes in variants performed by young chimpanzees certainly seem more consistent with a model in which "influenced" handclasps are the ones that have an effect on the partner's preferences.

Note also that while we had 21 individuals with GHC data at the age of ≤8 years old, only a small subset of these were informative for the model. To influence the model fit, we require a decent number of GHCs within one and the same observation period, with some potentially "influenced" events (e.g., see Figure S4). Examining such plots for all 21 individuals it became clear that we have only 3-5 individuals informing the model fit. Thus, the findings related to this hypothesis should be taken to mean that the patterns exhibited by a few individuals are consistent with the hypothesis of social transmission presented here. Further detailed data collected on chimpanzees of this age may require more definite conclusions to be drawn about the pathways of social transmission.

Figures

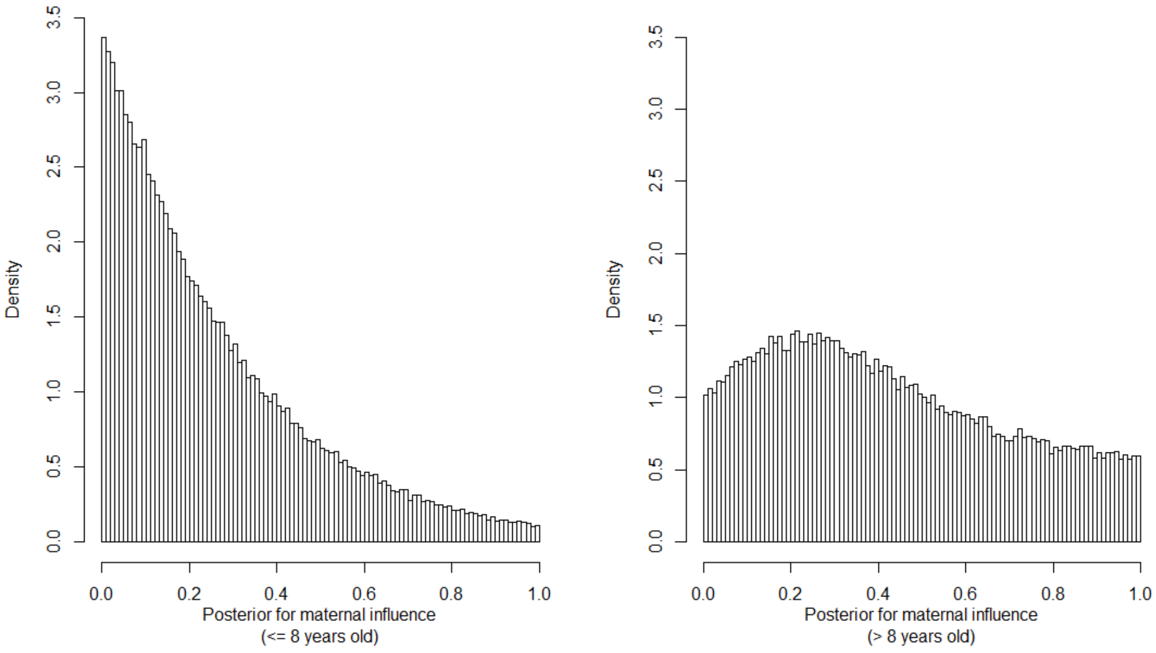

Fig. S1. Posterior distribution for effect of maternal influence on handclasp style preferences of chimpanzees of 8 years or younger (left) and of >8 years old (right).

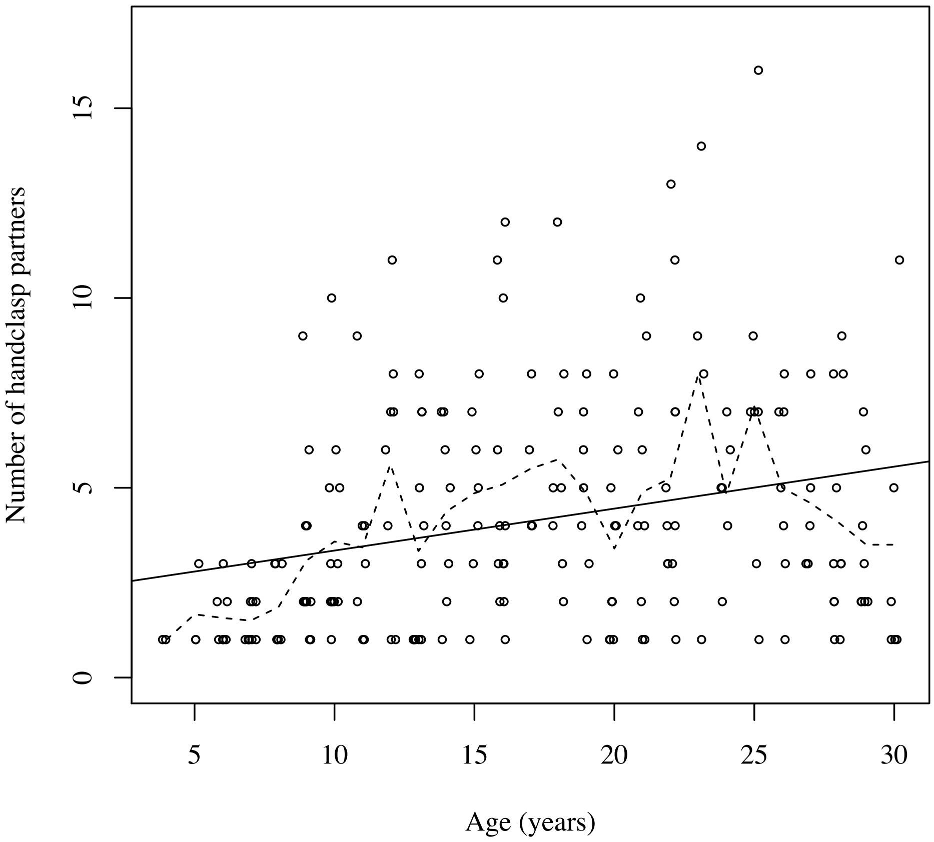

Fig. S2. Absolute number of unique handclasp partners (*y*-axis) as a function of subjects' age (*x*-axis). Each circle represents one individual, the dotted line follows the best smoothed model fit, the linear model-predicted trend is shown as solid line.

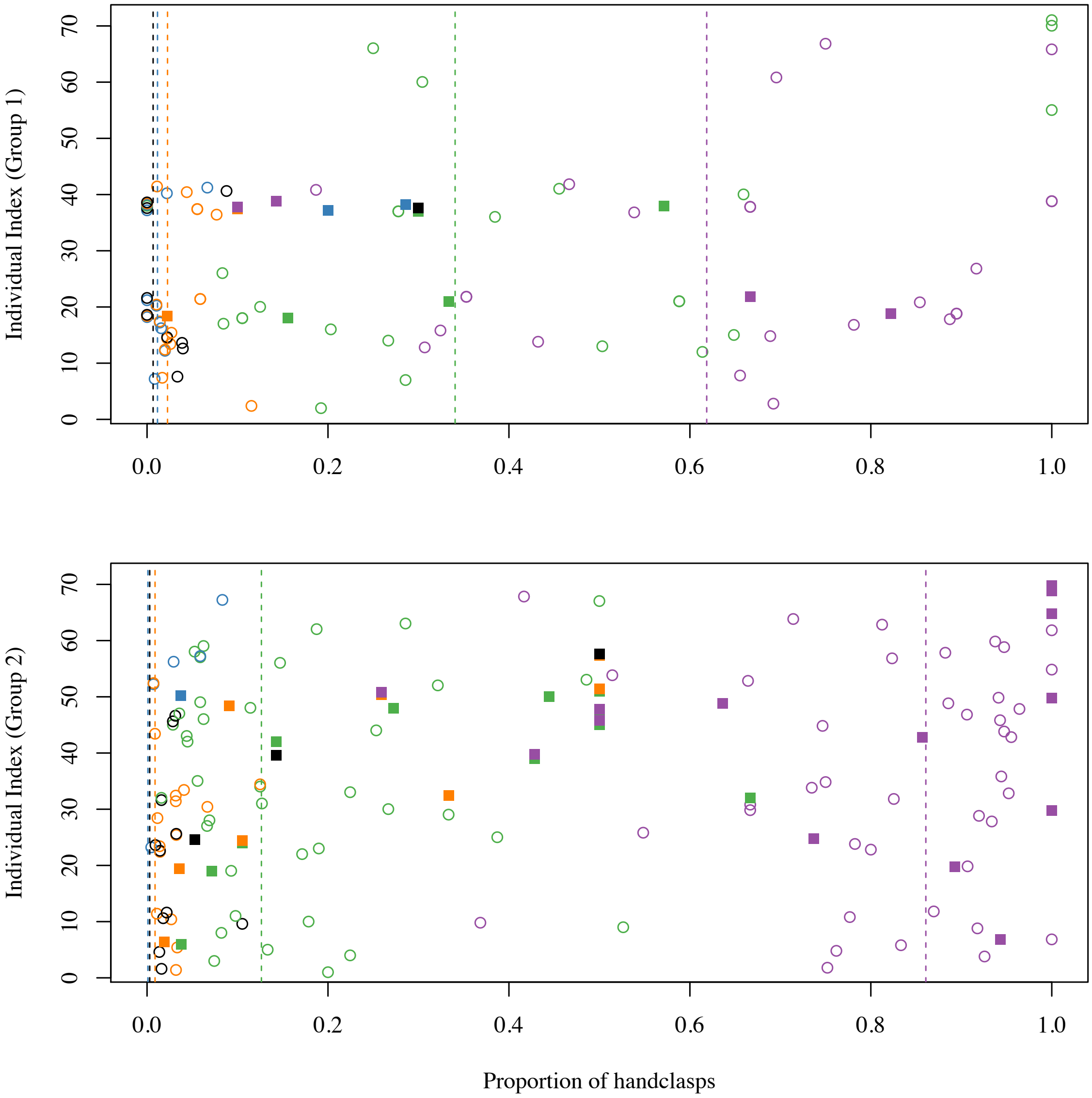

Fig. S3. The raw proportions of each option performed by each individual (green= wrist, black= elbow, yellow= forearm, blue= other and purple= palm). Dashed lines show the expected group preference $\hat{p}_{2k}$. Open circles are raw data for individuals >8 years old and filled squares are for individuals 8 years or younger. The points for younger individuals are more widely scattered around the group means than those for older individuals.

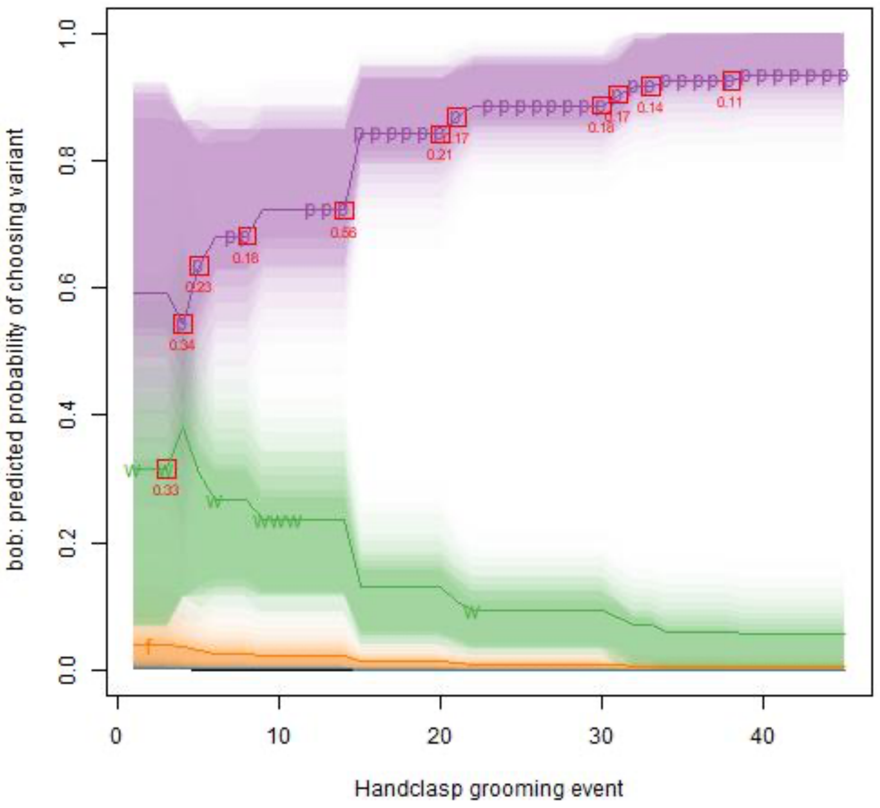

Fig. S4. Example of the pattern of handclasp events performed by an individual (Bob) when young (≤8 years old). p= palm variant, w= wrist variant, f= forearm variant. Red boxes show events in which the variant performed was potentially influenced (probability shown in red). The model-predicted preferences for each variant are shown as lines (median posterior) with the shaded section showing the density of the posterior distribution for each point (purple = palm; green= wrist; yellow= forearm). The pattern shown here is consistent with the hypothesis that young chimpanzees change their variant preference after engaging in GHC with influential partners because each potentially influenced event seems to result in an increase that the variant will be chosen by the subject (Bob – Group 1) in future handclasps.
